# Newcastle Disease Virus Induced Pathologies Severely Affect the Exocrine and Endocrine Functions of the Pancreas in Chickens

**DOI:** 10.3390/genes12040495

**Published:** 2021-03-29

**Authors:** Zaib Ur Rehman, Shanhui Ren, Salman Latif Butt, Zahid Manzoor, Javid Iqbal, Muhammad Naveed Anwar, Yingjie Sun, Xusheng Qiu, Lei Tan, Ying Liao, Cuiping Song, Weiwei Liu, Chunchun Meng, Chan Ding

**Affiliations:** 1 Shanghai Veterinary Research Institute (SHVRI), Chinese Academy of Agricultural Sciences (CAAS), Shanghai 200241, China; zaib.rehman@uaar.edu.pk (Z.U.R.); renxiaodeng988@163.com (S.R.); dr.naveed903@gmail.com (M.N.A.); sunyingjie@shvri.ac.cn (Y.S.); xsqiu1981@shvri.ac.cn (X.Q.); tanlei@shvri.ac.cn (L.T.); liaoying@shvri.ac.cn (Y.L.); scp@shvri.ac.cn (C.S.); liuweiwei@shvri.ac.cn (W.L.); 2 Faculty of Veterinary and Animal Sciences, PMAS Arid Agriculture University, Rawalpindi 46300, Pakistan; drzahidmanzoor@gmail.com; 3 UAF Sub Campus Toba Tek Singh, University of Agriculture, Faisalabad 36050, Pakistan; drsalman@uaf.edu.pk (S.L.B.); javed149@yahoo.com (J.I.); 4 Jiangsu Co-Innovation Center for Prevention and Control of Important Animal Infectious Disease and Zoonoses, Yangzhou University, Yangzhou 225009, China

**Keywords:** Newcastle disease virus, chicken, pancreas, hormone, enzymes, histopathology

## Abstract

Newcastle disease virus (NDV) causes a highly contagious and devastating disease in poultry. ND causes heavy economic losses to the global poultry industry by decreasing the growth rate, decrease in egg production high morbidity and mortality. Although significant advances have been made in the vaccine development, outbreaks are reported in vaccinated birds. In this study, we report the damage caused by NDV infection in the pancreatic tissues of vaccinated and specific-pathogen-free chickens. The histopathological examination of the pancreas showed severe damage in the form of partial depletion of zymogen granules, acinar cell vacuolization, necrosis, apoptosis, congestion in the large and small vessels, sloughing of epithelial cells of the pancreatic duct, and mild perivascular edema. Increased plasma levels of corticosterone and somatostatin were observed in NDV-infected chicken at three- and five- days post infection (DPI). A slight decrease in the plasma concentrations of insulin was noticed at 5 DPI. Significant changes were not observed in the plasma levels of glucagon. Furthermore, NDV infection decreased the activity and mRNA expression of amylase, lipase, and trypsin from the pancreas. Taken together, our findings highlight that NDV induces extensive tissue damage in the pancreas, decreases the activity and expression of pancreatic enzymes, and increases plasma corticosterone and somatostatin. These findings provide new insights that a defective pancreas may be one of the reasons for decreased growth performance after NDV infection in chickens.

## 1. Introduction

Newcastle disease (ND) is a highly infectious and one of the most distressing viral diseases in poultry, leading to heavy economic losses to the global poultry industry [1]. ND is caused by virulent strains of avian orthoavulavirus-1, also known as Newcastle disease virus (NDV) [2]. NDV belongs to the order *Mononegavirales*, family *Paramyxoviridae*, and genus *Orthoavulavirus*, containing a non-segmented, negative-sense, and single-stranded RNA genome of around 15 kilobases (kb) in length [3]. The proteins encoded by the NDV genome are nucleoprotein (NP), phosphoprotein (P), matrix (M), fusion (F), haemagglutinin-neuraminidase (HN), and large polymerase (L) [4]. The RNA editing of the P protein can result in the production of additional nonstructural proteins V and possibly W in the virus-infected cells [5,6]. From these proteins, F and HN are mainly involved in the NDV-induced pathologies in poultry.

There are four pathotypes of NDV strains based on clinical signs: asymptomatic enteric, lentogenic, mesogenic, and velogenic [7]. Mesogenic and velogenic strains of are responsible for systemic infections in poultry and clinical manifestations of variable mortality from subclinical infections to 100%. ND mainly affects the digestive, respiratory, and nervous systems [8,9,10,11] but is not limited to these organs. NDV-induced pathologies and replication have been noted in the spleen, liver, bursa of Fabricius, heart, and reproductive system [12,13,14,15,16]. Previous outbreaks of NDV in vaccinated and nonvaccinated poultry reported the involvement of the pancreas [15,17,18].

For the digestion of lipids, proteins, carbohydrates, and nucleic acids, different enzymes are produced by the digestive system, including the salivary glands, intestine, liver, and pancreas [19]; pancreatic enzymes such as trypsinogen, chymotrypsinogen, elastase, and procarboxypeptidase for the digestion of proteins; pancreatic amylase for the digestion of carbohydrates; phospholipase, lipase, and cholesterol esterase for the digestion of lipids; and two types of nucleolytic enzymes, namely ribonuclease and deoxyribonuclease [19,20,21,22]. The endocrine pancreatic tissue has clusters of cells known as islets of Langerhans that produce different hormones, including insulin, glucagon, somatostatin, and avian pancreatic polypeptide (APP) [23]. These hormones regulate glucose homeostasis, glucose and amino acid transport, lipogenesis, glycogenolysis, hypoglycemia, the level of plasma free fatty acid, the expression of lipogenic enzymes, intestinal motility, and gallbladder secretions [23].

Previous studies have shown the involvement of the pancreas in NDV infection, and histopathological lesions were reported in chickens [15,17,24,25], ducks [2,26], geese [27], and turkeys [28]. However, the disruption of the endocrine and exocrine functions of the pancreas is not clear in NDV-infected chickens. Our hypothesis was that chickens administered a smaller vaccine dose can suffer from pancreatitis, which may lead to a decrease in the production of the digestive enzyme. Therefore, the present study planned to investigate the pathophysiology of pancreatic functions in NDV-infected specific-pathogen-free (SPF) and vaccinated, and then NDV-challenged, chickens.

## 2. Materials and Methods

### 2.1. Virus

A wild-type velogenic NDV isolate, ZJ1 (Goose/China/ZJ1/2000; GB AF431744.3), was provided by Professor Xiufan Liu from Yangzhou University (Yangzhou, China). The pathogenicity indices, including mean death time (MDT), intracerebral pathogenicity index (ICPI), and intravenous pathogenicity index (IVPI), of the ZJ1 were 51.6, 1.89, and 2.7, respectively [27,28]. The NDV lentogenic strain LaSota was obtained from the China Institute of Veterinary Drug Control (Beijing, China). The virus stock was prepared by growing the virus in 10-day-old SPF-embryonated chicken eggs, and harvested allantoic fluid was subsequently stored at −80 °C until further use.

### 2.2. Chickens, Ethics Statement, and Experimental Design

All the animal experimental procedures were performed in strict accordance with the recommendations in the Guide for the Care and Use of Laboratory Animals of Shanghai Veterinary Research Institute (SHVRI, Shanghai, China) of the Chinese Academy of Agricultural Sciences (CAAS, Beijing, China). All protocols applied in this study were approved by the Institutional Animal Care and Use Committee of SHVRI (Permission Number: SHVI-RO-2018110123), CAAS, China.

Specific-pathogen-free embryonated chicken eggs were purchased from the Merial Vital Laboratory Animal Technology Company (Beijing, China) and were incubated at the laboratory facility of SHVRI, CAAS. The hatched-out SPF chicks were reared, vaccinated, and challenged in positive-pressure isolators in a high containment facility. All the birds were provided with ad libitum access to feed and water throughout the experiment. At the age of 9 weeks, chickens were randomly divided into three groups having 20 chickens each.

Birds in Group 3 (vaccinated challenged (VAC + CHA)) were vaccinated at the age of 9 weeks with 100 µL of La Sota at the dose rate of 10^3^ embryo infective dose (EID_50_/0.1 mL) via intranasal and eye drop routes, prepared in phosphate-buffered saline (PBS). After 2 weeks of vaccination of the VAC + CHA group, all the birds in Group 2 (CHA = nonvaccinated challenged) and the VAC + CHA group were challenged with ZJ1 suspended in PBS (stored at 4 °C overnight) having a titer of 10^6.5^ EID^50^/0.1 mL per bird via the right eye and a choanal slit at a 100 µL volume. Doses of the challenged and vaccinated viruses were selected on the basis of the previous experiment of Cornax et al. [29]. Birds in Group 1 (control (CON)) were given the same volume of PBS, via the same route, as the mock infection.

### 2.3. Sampling

At 1, 3, and 5 days postinfection (DPI), blood was collected in EDTA-coated tubes (Shijiazhuang Kang Weishi Medical Instrument Co., Ltd., Shijiazhuang, China), and the chickens were euthanized by intravenous injection of sodium pentobarbital in the brachial vein for the collection of tissue (pancreas) samples. Blood and tissue samples were collected from four birds per group and immediately transferred to the laboratory, maintaining the cold chain. To obtain the plasma, blood samples were centrifuged (2000× *g*) for 10 min at 4 °C and stored at −80 °C until further analysis. All the tissue samples were immediately put in liquid nitrogen and stored at −80 °C until further analysis.

### 2.4. Plasma Hormones

Plasma levels of the somatostatin, insulin, and glucagon were determined using chicken-specific commercial assay kits (Nanjing Jiancheng Bioengineering Institute (HO92-SS; H183 GC; H203 Insulin), Nanjing, China). All samples were analyzed within a single assay to avoid interassay variations. The absorbance of the microplates was determined using the Epoch microplate spectrophotometer (BioTek Instruments, Inc., Winooski, VT, USA) at the optical density value of 450 nm. The plasma levels of corticosterone (CORT) were measured using a chicken-specific commercial ELISA kit (CSB-E11991C; Cusabio Biotech. Co., Ltd., Wuhan, China). The standard curve was created using the CurveExpert 1.4. The limits of detection for the insulin and glucagon were 0.5 mIU/mL and 5 ng/L, respectively. The inter- and intra-assay coefficient of variation was less than 10% and 12%, respectively.

### 2.5. Digestive Enzyme Activities in the Pancreas

To determine the activity of the pancreatic enzymes, a small portion (approximately 1 mg) of the pancreatic tissue was homogenized in 9 volumes (*w*/*v*) of ice-cold phosphate-buffered saline (PBS) using a Tissuelyser-24 (Jingxin Technology, Shanghai, China) at 20 Hz for 2 min. The homogenate was instantly centrifuged at 3000× *g* at 4 °C for 10 min, and the supernatant was collected for the determination of digestive enzyme activities. To determine the activity of trypsin (A080), lipase (A054), and amylase (C016) by calorimetric methods, commercial kits were used (Nanjing Jiancheng Bioengineering Institute, Nanjing, China). Pre-experimental standardizations were performed for each of the targeted enzymes to determine the optimum dilution for the measurement of enzymes. Protein concentrations of the supernatants were determined using the kits from Beyotime, (Beyotime, Nanjing, China; P0010).

### 2.6. Quantitative Real-Time PCR

Quantitative real-time PCR (qRT-PCR) was performed to measure the mRNA expressions of pancreatic digestive enzymes, including amylase, lipase, and trypsin genes. The chicken glyceraldehyde 3-phosphate dehydrogenase (GAPDH) was used as an internal control. The primers used in the present study are described in Table S1. Total RNA of the pancreatic tissue was extracted using TRIzol reagent (Invitrogen, Carlsbad, CA, USA) and resuspended in RNase-free water. Reverse transcription was performed using HiScript II (Catalog Number: R233; Vazyme Biotech Co., Ltd., Nanjing, China), according to the manufacturer’s instructions. Expression of target genes was measured by performing qRT-PCR on the CFX96 Touch Real-Time PCR Detection System (BioRad, Hercules, CA, USA) using SYBR Premix (Dongsheng Biotech, Guangzhou, China), and data were normalized with GAPDH (internal control). qRT-PCR was performed with a final volume of 20 µL. The PCR cycles are as follows: 94 °C for 3 min, followed by 40 cycles of 95 °C for 15 s, 60 °C for 15 s and 72 °C for 20 s (For primer sequence see Table S1). All the amplifications generated expected amplicons with single, sharp fusion curves. All experiments were carried out in triplicate. The changes of mRNAs were presented as fold expression and calculated using the 2^−ΔΔCT^ method [30].

### 2.7. Viral Loads in the Pancreas

The virus copy numbers were detected from the pancreas, as described previously [12]. Briefly, the ZJ1 strain was grown in 10-day-old SPF-embryonated eggs, and allantoic fluid was collected after 60 h of infection. The TRIzol (Invitrogen, Carlsbad, CA, USA) reagent was used to extract the viral RNA from the allantoic fluid as per the manufacturer’s instructions. The ZJ1 M gene was amplified using the primers mentioned in Table S1, and the size (1095 base pair) of the fragment was confirmed by electrophoresis. The HiPure Gel Pure DNA Mini Kit (AnGen Biotech, Guangzhou, China) was used to purify the PCR product and cloned into a plasmid vector to construct a standard curve. The pancreatic tissue from the chickens was homogenized, and RNA was extracted as mentioned above. About 1 μg RNA extracted from the pancreas samples was reverse transcribed to cDNA with HiScript II (Catalog Number: R233; Vazyme Biotech Co., Ltd., China), and quantitative PCR was performed with the SYBR Premix (Dongsheng Biotech, China). Virus copy numbers were calculated using the standard curve [12].

### 2.8. Histopathology

Samples of the pancreatic tissue were collected from the middle region and fixed in 10% neutral-buffered formalin, embedded in paraffin, and 5 µm thick sections were obtained. The sections were stained with hematoxylin and eosin (H&E) following the standard procedures [31]. The slides were digitalized using Pannoramic SCAN (3DHISTECH Ltd., Budapest, Hungary), and histological lesions were studied using CaseViewer 2.2 (3DHISTECH Ltd., Hungary) [32].

### 2.9. TUNEL Assay

For the detection of apoptotic cells, 5 mm thick tissue sections of the pancreatic tissue were subjected to the transferase dUTP nick-end labeling (TUNEL) assay according to the manufacturer’s instructions (Roche Diagnostics GmbH, Mannheim, Germany). Tissue sections were deparaffinized and rehydrated by the graded levels of xylene and ethanol. Tissue sections were treated with proteinase K and incubated at 37 °C for 25 min. The activity of the endogenous peroxidase activity was blocked with hydrogen peroxide. The sections were incubated at 37 °C with the terminal TdT nucleotide mixture for 2 h. Nuclei were counterstained with 3 µg/mL 4′ and 6′-diamidine-2-phenylindole (DAPI) and visualized by fluorescence microscopy. Slides were then digitalized using Pannoramic SCAN (3DHISTECH Ltd., Hungary) and studied using CaseViewer 2.2 (3DHISTECH Ltd., Hungary). Five different high power fields (HPFs) were selected to count the number of apoptotic cells using Image-Pro Plus software (version 6.0 for Windows) (Media Cybernetics, Rockville, MD, USA).

### 2.10. Statistical Analysis

The data were analyzed by a two-way ANOVA test, with challenge and time points as the main effects [33]. The graphical results were expressed as mean ± standard deviation (M ± SD). Results with *p* < 0.05 were considered statistically significant. When a significant main effect was observed, Tukey’s test was used to compare the differences among groups. Graph Pad Prism 6.0 software (GraphPad Software, Inc., San Diego, CA, USA) was used to generate the graphs.

## 3. Results

### 3.1. Viral Load in the Pancreas

**Figure 1:**
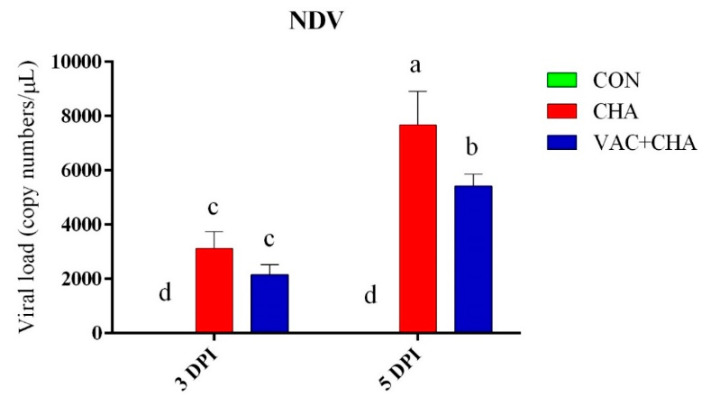
Relative quantity of the NDV-M gene in the pancreas of chicken. The bars represent the standard deviation of the mean. Columns without a common superscript letter differ significantly (*p* < 0.05) in the figure. CON, control; CHA, Newcastle disease virus challenged; VAC + CHA, vaccinated with LaSota and challenged with virulent Newcastle diseases virus after 2 weeks of vaccination. The NDV-M gene was not detected in the control group.

The transcriptional level of the NDV-M gene was determined to examine the viral load in the pancreas using the qRT-PCR assay at 3 and 5 DPI. The viral copy numbers per µL are shown in Figure 1. There was no significant difference in the viral load of the VAC + NDV and CHA groups at 3 DPI. The viral copy numbers were significantly less at 3 DPI compared to 5 DPI in the CHA and VAC + CHA groups. Maximum viral load observed in the CHA group at 5 DPI was significantly higher compared to the VAC + NDV group. The negative control group remained negative for NDV.

### 3.2. Histopathology

**Figure 2:**
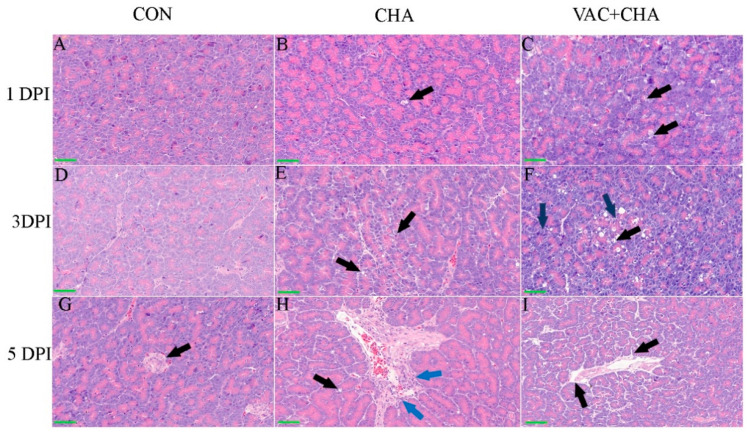
Histopathological changes induced by the Newcastle disease virus (ZJ1) in the pancreas of specific-pathogen-free (SPF) and vaccinated chicken. Panels (**A**–**C**), (**D**–**F**), and (**G**–**I**) show the histopathology of the control, NDV-infected (CHA), and vaccine + NDV (VAC + CHA)-infected groups at 1-, 3-, and 5-days post infection (DPI), respectively. Histopathological changes were not found from the control group. Severe histopathological changes were induced by the NDV infection in SPF chickens, including (**B**) single-cell, (**E**) cytoplasmic vacuolation, and single-cell necrosis (arrow) and (**H**) single-cell necrosis (black arrow) and perivascular lymphocytic infiltration (blue arrows). However, in vaccinated chickens, histopathological changes induced by the NDV challenge include (**C**) individualized shrunken cells. (**F**) The black arrow shows single-cell necrosis, and the blue arrow shows individualized and shrunken cells and (**I**) mild perivascular edema at 5 DPI. Panels in the control group do not show any pathological changes, and unaffected endocrine pancreatic cells from the control group (**G**) are marked with arrowheads.

There were no significant changes observed in the control group. At certain locations, the presence of intranuclear eosinophilic material could be nuclear edema or nuclear invagination or an incidental finding. In the CHA group, at 1 DPI, the pancreas was intact. Multifocal lymphoid nodules in the proximity of branches of the pancreatic ducts and, occasionally, hyperplasia in the endocrine pancreas were seen. Rarely, cells in the acinar tissue had an eosinophilic intranuclear material. This was only observed in the endocrine tissues. There was mild perivascular edema. Rare multifocal apoptotic cells (rounded, hypereosinophilic with pyknotic nuclei) were noticed. At 3 DPI, in the CHA group, eosinophilic intranuclear material was noticed in the endocrine pancreas. Multifocal to diffusely, there was partial depletion of zymogen granules from the exocrine pancreas. Acinar cells had occasional single to multiple vacuoles in the apical portion of the cell. Multifocally, there was mild perivascular edema and, occasionally, the individualization of acinar cells. Few acinar cells underwent single-cell necrosis/apoptosis, and other cells had mild cytoplasmic vacuolation. No significant changes were observed in the endocrine pancreas. Occasionally, there was lymphocytic infiltration in the exocrine tissue. Rarely, there were eosinophilic intranuclear inclusion bodies in the endocrine tissue (Figure 2E). At 5 DPI, in the CHA group, exocrine cells had a complete loss of zymogen granules, which rounded off and separated from neighboring cells. The exocrine and endocrine tissues lost their original structure, and cells were individualized. There was severe congestion in the large and small vessels. The epithelial cells of the pancreatic duct were sloughed and also lost nuclear detail. There was mild perivascular edema. Occasionally, there was single-cell necrosis (Figure 2H). There was cytoplasmic vacuolation of acinar cells in the exocrine tissue and partial depletion of zymogen granules.

In the VAC + CHA group, at 1 DPI, the pancreatic tissues had, multifocal, partial to complete depletion of zymogen granules, cytoplasmic vacuolation (degeneration), and sometimes necrosis of individual acinar cells of the pancreas. At 3 DPI, the acinar cells underwent a spectrum of changes that ranged from degeneration, with the loss of zymogen granules and cytoplasmic vacuolation, and swelling of the cells, to single-cell necrosis, with cells becoming individualized and shrunken with pyknotic nuclei. There were multifocal, randomly distributed lymphoid nodules, whereas, at 5 DPI, partial or complete depletion of zymogen granules was observed. Frequently, cytoplasmic vacuolation with eosinophilic hyaline inclusion in acinar cells of the pancreas was seen throughout the exocrine part. There were multifocal lymphoid nodules throughout the exocrine pancreas. There was a marked increase in the cytoplasmic vacuolation and single-cell necrosis of acinar cells, and due to the partial depletion of zymogen granules, acinar cells looked ductless. There was mild to moderate interstitial edema in the exocrine portion of the pancreas and the separation of acinar cells from neighboring cells. One focal area of lymphocytic infiltration in the peripancreatic duct branch, perivascular edema, was observed. Persistently, scattered lymphocytes were observed throughout the exocrine pancreas. Most of the observed lesions were perivascular in this slide (Figure 2I). Individualized round acinar cells with dark pyknotic nuclei showed severe single-cell necrosis throughout the exocrine tissue. There was a distortion of the acinar cells and separation from the neighboring cells. Diffusely, the areas of cytoplasmic vacuolation of acinar cells are seen.

### 3.3. Apoptosis in the Pancreas

**Figure 3:**
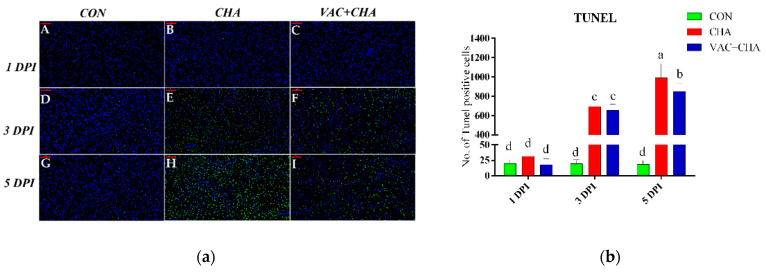
(**a**) T Fluorescent micrographs of chicken pancreas infected with NDV and stained by TUNEL assay technique. Panels (**A**,**D**,**G**) represent the control (CON) group at 1-, 3- and 5-days post infection (DPI). Panels (**B**,**E**,**H**) represent the NDV-challenged (CHA) SPF chickens. Panel (**C**,**F**,**I**) represent the vaccinated and NDV (VAC + CHA) challenge birds. Apoptotic cells show green nuclei, whereas the nuclei in the normal cells are shown in blue color. (**b**) The graphical representation of TUNEL positive cells in different groups. The green, red, and blue columns represent control, nonvaccinated challenged, and vaccinated challenged groups, respectively. The bars represent the standard deviation of the mean and lower-case letters show statistical significance (*p* < 0.05) among different groups. CON, control; CHA, Newcastle disease virus challenged; VAC + CHA, vaccinated with LaSota and challenged with virulent Newcastle diseases virus after 2 weeks of vaccination.

To estimate NDV-induced apoptosis in the chickens’ pancreatic tissue, TUNEL-positive cells were counted (Figure 3). The apoptotic cells had green-stained nuclei. As presented in Figure 3a, the number of apoptotic-positive cells in the pancreas was similar between the CON- and NDV-infected groups at 1 DPI. In the NDV-challenged groups, the number of apoptotic cells was significantly increased compared to CON at 3 DPI. The increase in the number of apoptotic cells was less evident in the VAC + NDV group compared to CHA; however, it remained significantly higher than the CON group (Figure 3b) at 5 DPI.

### 3.4. Plasma Concentrations of Hormones

**Figure 4:**
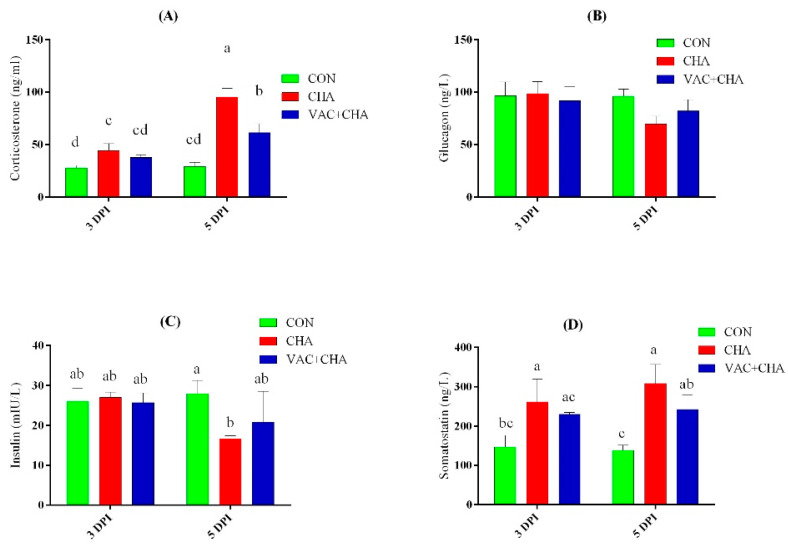
Mean levels of the plasma corticosterone (**A**), glucagon (**B**), insulin (**C**), and somatostatin (**D**) at 3 DPI and 5 DPI in vaccinated and nonvaccinated chickens challenged with NDV (ZJ1). The green, red, and blue columns represent control, nonvaccinated challenged, and vaccinated challenged groups, respectively. The bars represent the standard deviation of the mean and lower-case letters show statistical significance (*p* < 0.05) among different groups. CON, control; CHA, Newcastle disease virus challenged; VAC + CHA, vaccinated with LaSota and challenged with virulent Newcastle diseases virus after 2 weeks of vaccination.

The plasma concentrations of CORT were significantly elevated (*p* < 0.05) in the CHA group compared to CON at 3 DPI. A significant difference was not found in the concentrations of CORT in CON and VAC + CHA (Figure 4A). However, at 5 DPI, the plasma CORT level in the CHA and VAC + CHA groups was significantly higher compared to the CON group.

Plasma concentrations of glucagon and insulin are represented in Figure 4B, C, respectively. Statistically, no significant effect of NDV challenge on glucagon was observed at 3 and 5 DPI, but numerically, a slight decrease in the plasma levels of NDV-infected birds was noticed compared to the CON group. The plasma levels of insulin remained unaffected at 3 DPI, but a significant decrease was observed in CHA group compared to the control at 5 DPI (Figure 4C).

The plasma concentration of the somatostatin is presented in Figure 4D. The plasma concentrations of the somatostatin were significantly increased (*p* < 0.05) in the CHA (from 261114c) group, but this increase was not significant in the CON and VAC + CHA groups, at 3 DPI. Birds in the CHA group tend to have a higher concentration (*p* < 0.05) of somatostatin at 5 DPI compared to the CON and VAC + CHA groups.

### 3.5. Activity and Expression of Pancreatic Enzymes

**Figure 5:**
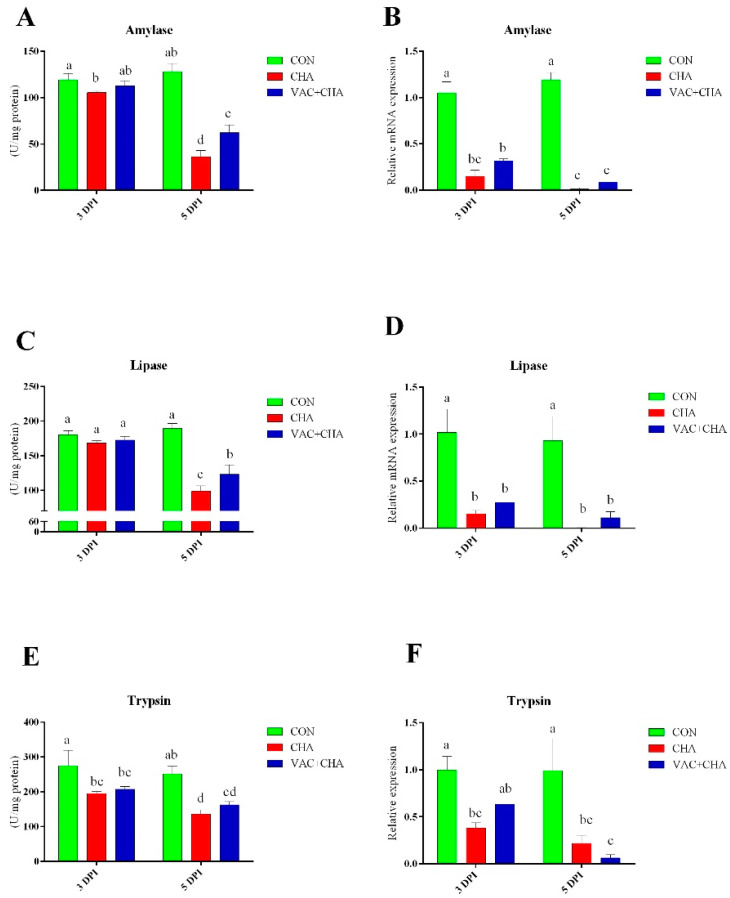
Mean level of activity and relative expression of the genes of pancreatic enzymes; Amylase (**A**,**B**), lipase (**C**,**D**) and trypsin (**E**,**F**) at 3 and 5 DPI in vaccinated and nonvaccinated chickens challenged with NDV (ZJ1). The green, red, and blue columns represent control, nonvaccinated challenged, and vaccinated challenged groups, respectively. The bars represent the standard deviation of the mean, and the lower-case letters show statistical significance (*p* < 0.05) among the different groups. CON, control; CHA, Newcastle disease virus challenged; VAC + CHA, vaccinated with LaSota and challenged with virulent Newcastle diseases virus after 2 weeks of vaccination.

The results for amylase activity and expression are presented in Figure 5A,B. At 3 DPI, chickens in the CHA group showed decreased activity of amylase (*p* < 0.05), compared to the CON group. However, amylase activity in the pancreas of the VAC + CHA group of chickens was similar to that observed in the CON group at 3DPI. More drastically a decrease in the activity and expression of amylase in the pancreas of the CHA groups of chickens was observed at 5 DPI compared to the CON and VAC + CHA groups. Furthermore, the activity of amylase in the VAC + CHA group was significantly less than the control and more than the CHA group. The expression of amylase at 5 DPI from the CHA and VAC + CHA groups was significantly (*p* < 0.05) less than the CON group.

There was no significant difference in the activity of lipase at 3 DPI (Figure 5C). NDV infection in SPF chickens resulted in the decreased activity of lipase compared to chickens in the CON and VAC + CHA groups. Lipase mRNA expression in the pancreas significantly decreased in NDV-infected birds (*p* < 0.05) compared to CON at 3 and 5 DPI (Figure 5D).

The NDV challenge significantly affected the activity and expression of pancreatic trypsin (Figure 5E,F). The activity of trypsin was significantly decreased in the NDV-challenged groups compared to CON, and this decrease was more obvious at 5 DPI. The mRNA expression of trypsin was significantly higher in the pancreas of CON birds (*p* < 0.05) compared to the CHA and VAC + CHA groups.

## 4. Discussion

The present study comprehensively reported the effect of NDV infection on the pancreas in vaccinated and SPF chickens. We persuasively demonstrated the replication of NDV in the pancreas of vaccinated and SPF chickens. In the study, chickens were vaccinated with 10^3^ EID50 of the La Sota and challenged with 10^6.5^ EID50 of the ZJ1 after two weeks of vaccination because, at this dose of vaccine and challenge, the pathogenic virus can replicate and cause conjunctivitis and mild depression [29]. Histological studies of the chickens in the current experiment advocate severe damage caused by NDV in the pancreas. NDV infection caused the partial depletion to complete loss of zymogen granules from the exocrine pancreas, cytoplasmic vacuolation, and necrosis of acinar cells, and eosinophilic intranuclear material was noticed in the endocrine pancreas, but these were less evident at 3 DPI compared to 5 DPI. The histopathological results of the present study are in agreement with previous studies [15,17,24,25,26].

NDV infection in vaccinated and SPF chickens resulted in a significant decrease in the activities and expressions of pancreatic enzymes. These decreased activities and expressions of amylase, lipase, and trypsin may be related to extensive damage caused by NDV to the exocrine portion of the pancreas in the infected birds. These decreased activities of the pancreatic enzymes may be among the reasons for the poor feed conversion ratio in NDV-infected vaccinated birds [34]. Decreased activities of the digestive enzyme may be related to poor production performance after NDV outbreaks in commercial poultry.

It is well studied that the hypothalamic–pituitary–adrenal axis is involved in acute stress and regulates the inflammatory response to an infectious challenge. Stress is strongly correlated with the exacerbation of many viral infections [35,36]. In this study, increased plasma levels of corticosterone were found in NDV-infected chicken. Increased levels of corticosterone have been related to a decreased growth rate, decreased antibody response, decreased number of lymphocytes, and reduced size of lymphoid organs [37]. Corticosterone decreases insulin sensitivity in chickens [38]. In this study, a slight decrease in the plasma levels of insulin and glucagon was noted at 5 DPI in NDV-infected chickens. This may be due to fewer structural and morphological changes in the endocrine pancreas after NDV infection, as noted in the histopathology results. The avian pancreas has higher levels of glutathione, which exhibits protective effects on the β cells [39]. This increased level of glutathione may be related to less pathogenicity in the endocrine pancreas because decreased levels of glutathione were observed in the plasma and intestines of NDV-infected chickens [9,40].

Previous studies have suggested the production of somatostatin (SS) from endocrine cells in the proventriculus, small intestine [41], and pancreas [42], as well as expression in the testis [43]. There are two forms of chicken somatostatin, somatostatin-14 and somatostatin-28, which are encoded by the same gene (PSS1), but another variant exists that is expressed in the brain and pancreatic islets [23,44]. The somatostatin inhibits the production of many hormones, including insulin, glucagon, growth hormone, gastrin, vasoactive intestinal peptide, and thyroid-stimulating hormone [45]. In the present study, increased plasma levels of SS were found in NDV-challenged birds. Increased somatostatin mRNA expression has been described after stimulation with cytokines in murine macrophages [46]. Similarly, the increased plasma concentration of somatostatin in endotoxin-injected sheep [47], septic pigs [48], and simian immunodeficiency virus-infected macaque, then infected with the Mycobacterium avium [49], increased expression in the rabies-virus-infected brain [50] and secreted by human adipocytes after stimulation with activated macrophages, LPS, and IL-1β [51]. The increased plasma concentrations of somatostatin may be involved to counter the inflammation caused by infection. The SS has been described as anti-inflammatory and immunosuppressive, and its analogs have been used as potential treatments for inflammation [52,53].

## 5. Conclusions

In summary, the present study suggests that the pancreas could be a potential target of NDV infection. Evidently, the exocrine pancreas is more severely affected by NDV compared to the endocrine pancreas. NDV infection leads to a decrease in the production and activity of digestive enzymes from the pancreas, even in vaccinated chickens. Similarly, NDV infection also led to increased levels of corticosterone and somatostatin and decreased production of insulin, as evident from the plasma levels. These pathologies of the pancreas may be the leading cause of the decreased production performance of NDV-infected vaccinated birds.

## Data Availability

Data supporting the conclusions of this article are included within the article.

## Supplementary Materials

The following are available online at https://www.mdpi.com/article/10.3390/genes12040495/s1, Table S1: List of primer sequences used for quantitative PCR.
