# Supplementary material for: Newcastle Disease Virus Induced Pathologies Severely Affect the Exocrine and Endocrine Functions of the Pancreas in Chickens
Source: Genes (Basel). 2021 Mar 29;12(4):495. doi: 10.3390/genes12040495 (PMC8067305; doi:10.3390/genes12040495)
Supplement: Supplementary file 1 [file genes-12-00495-s001.pdf]

Table S1. List of primer sequences used for quantitative PCR.

Gene Type	Forward Primer(5′-3′)	Reverse primer(5′-3′)	Amplicon size (base pair)	Gen Bank accession number
ZJ1 M gene	ATGGACTCATCCAGGACAATCGGGCT	TTATTTCCTGAAAGGATTGTATTTAGCAATGG	1095	AF431744.3
ZJ1 M gene	TACTTTGATTCTGCCCTCCCTT	TAAGCAGAGCATTGCGGAAGA	255	AF431744.3
Trypsin	GTCGCCTTTGTTGGTGTGAC	GCTGATGAGAGAGCCTCCAC	150	NM_205385
Lipase	AGGAGAAGAAGGCTGGACCT	TCAGCACCTACAACACGGAC	141	XM_015288675
Amylase	TCAGGCTGGGAGGACATCTA	CCAGGGCCTGTTCGGATTAG	157	NM_001001473
GAPDH	CCATCACAGCCACACAGAAGAC	TGGACGCTGGGATGATGTT	93	NM_204305
